# The *Salmonella* effector SseJ disrupts microtubule dynamics when ectopically expressed in normal rat kidney cells

**DOI:** 10.1371/journal.pone.0172588

**Published:** 2017-02-24

**Authors:** Sally A. Raines, Michael R. Hodgkinson, Adam A. Dowle, Paul R. Pryor

**Affiliations:** 1 Department of Biology, Wentworth Way, University of York, York, United Kingdom; 2 Technology Facility, Department of Biology, Wentworth Way, University of York, York, United Kingdom; 3 Hull York Medical School, University of York, York, United Kingdom; Centre National de la Recherche Scientifique, Aix-Marseille Université, FRANCE

## Abstract

*Salmonella* effector protein SseJ is secreted by *Salmonella* into the host cell cytoplasm where it can then modify host cell processes. Whilst host cell small GTPase RhoA has previously been shown to activate the acyl-transferase activity of SseJ we show here an un-described effect of SseJ protein production upon microtubule dynamism. SseJ prevents microtubule collapse and this is independent of SseJ’s acyl-transferase activity. We speculate that the effects of SseJ on microtubules would be mediated *via* its known interactions with the small GTPases of the Rho family.

## Introduction

*Salmonellae* are gram-negative bacteria that can infect a wide range of hosts and in humans can cause diseases such as typhoid fever and gastroenteritis. There are ~2600 recognized *Salmonella* serovars of which over half are represented by *S*. *enterica* subspecies *enterica* (*S*. *enterica* subspecies I), constituting 99% of human clinical *Salmonella* infections. *Salmonella enterica* serovar Typhimurium (*S*. Typhimurium; the cause of gastroenteritis) uses two type III secretion systems (T3SS) to translocate pathogen effector proteins directly into the host cell’s cytoplasm. (reviewed by [1]). The T3SS encoded by *Salmonella* pathogenicity island-1 (SPI-1; T3SS-1) is mostly active when extracellular *Salmonella* come into contact with a host cell and allows effector proteins to be translocated directly into the cell cytoplasm and causes the bacteria to be actively phagocytosed. Another T3SS encoded by *Salmonella* pathogenicity island-2 (SPI-2; T3SS-2) enables the bacteria to multiply intracellularly in a Salmonella containing vacuole (SCV) by allowing further effector proteins to be translocated directly from the *Salmonella* (through the phagosomal membrane) into the host cell cytoplasm. It is unclear precisely how *Salmonella* uses its multiple T3SS effector proteins to survive intracellularly but theories range from delaying fusion with the degradative organelle the lysosome [2], though the role of the T3SS in this process is contested [3], to preventing the delivery of lysosomal hydrolases to the *Salmonella*-containing phagosomal compartment by altering mannose 6-phosphate receptor trafficking [4]. Only a finite number of intracellular membrane trafficking and signalling events can be manipulated by a pathogen and hence successful intracellular pathogens are often found to target the same host cell molecules, for instance phosphoinositides are targeted by both *Salmonellae* and *Mycobacteria* [5, 6]. Understanding how *Salmonella* survives intracellularly not only provides information about *Salmonella* pathogenesis but potentially what processes may also be targeted by other intracellular pathogens.

To understand the role of *Salmonella* T3SS effector proteins in the flow of membranes to the lysosome a rapid screen was undertaken in *Saccharomyces cerevisiae (S*. *cerevisiae)*. Membrane trafficking events are conserved between yeast and mammalian cells. Therefore, yeast can be used to rapidly identify any *Sallmonella* proteins that alter membrane traffic to the yeast vacuole, the equivalent of the mammalian lysosome. The screen identified the *Salmonella* virulence protein SseJ and subsequently we show a previously un-described effect of this protein on the stability of host cell microtubules. Microtubules are required for phagosome fusion [7–9] and by promoting a network of stable microtubules this can aid in phagosome fusion with endocytic organelles enabling nutrients to be delivered to the phagosomal lumen, promoting bacterial replication.

## Results

### SseJ production causes membrane trafficking defects

**Fig 1:**
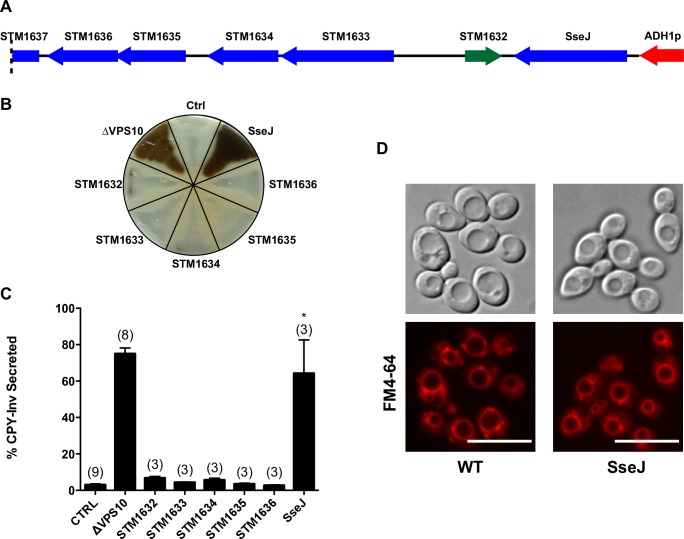
Expression *of ssej* causes CPY-Inv to be mis-sorted in *Saccharomyces cerevisiae*. (A) Fragment of the *Salmonella* chromosome inserted into the yeast expression vector causing CPY-Inv secretion. (B) Qualitative CPY-Inv secretion in yeast expressing individual *Salmonella* genes identified in (A). Negative control yeast (ctrl) contain just the cloning vector (pVT-100U) and positive control yeast lack the receptor VPS10 for CPY (ΔVPS10). (C) Quantitative CPY-Inv secretion in yeast expressing *Salmonella* genes. Controls as in (B). Data are from n = 3–9 (number of experiments for each condition in parentheses above each bar) and are mean ± S.D. *P<0.001 SseJ c.f. Ctrl (P>0.05 SseJ c.f. ΔVPS10). (D) Fluorescence visualisation of the yeast vacuole in wild type yeast (WT) transformed with vector (pVT-100U) alone or SseJ in pVT-100U (SseJ). Top panels DIC and bottom panels FM 4–64 fluorescence. Scale bar = 10 μm.

To identify *Salmonella* proteins that can disrupt intracellular membrane trafficking, a genomic library from *Salmonella* was generated and the DNA inserted into a yeast expression vector. *S*. *cerevisiae* were then transformed with the plasmid library and colonies screened for a defect in the delivery of the vacuolar hydrolase, carboxypeptidase-Y (CPY), to the yeast vacuole. If there is disruption of CPY delivery to the vacuole then CPY is secreted. We assayed the secretion of a CPY-invertase fusion protein that oxidises an applied solution of o-diansidine to a brown precipitate [10]. This approach has been successfully employed to identify effector proteins of *Legionella pneumophila* [11] and *Mycobacterium tuberculosis* [12] that interfere with yeast membrane trafficking. Yeast transformed with the plasmid library were screened for CPY-Inv secretion and 8 yeast clones were found to have CPY-Inv secretion in a plasmid dependent manner. One of the clones identified a 6kb fragment of *Salmonella* chromosomal DNA containing 1 partial open reading frame (ORF) and 6 complete ORFs (Fig 1A). All of the *Salmonella* genes identified in the plasmid, were cloned and expressed individually in yeast and re-assayed for CPY secretion. Qualitative CPY-Inv secretion on agar plates showed that SseJ caused CPY secretion, though we did not analyse the protein production levels of the other 5 proteins. (Fig 1B). Quantitative CPY-Inv secretion from yeast in liquid culture demonstrated that SseJ dependent CPY-Inv secretion was equivalent to that in yeast lacking the CPY receptor, VPS10 (ΔVPS10; Fig 1C). There are numerous intermediate vesicles involved in delivery of CPY to the vacuole and the retrograde trafficking of the VPS10 receptor. When CPY is secreted, due to a trafficking defect, it is possible to examine the phenotype of the yeast vacuole and in some cases determine which part of the trafficking step of CPY from the Golgi to the vacuole is disrupted [13, 14]. Using the membrane dye FM4-64 to label the yeast vacuole in yeast expressing SseJ, no differences in the morphology of the vacuole were seen compared to wild-type yeast (Fig 1D). These data indicated that SseJ alone can cause a membrane trafficking defect in yeast.

### SseJ production re-distributes late endocytic organelles

**Fig 2:**
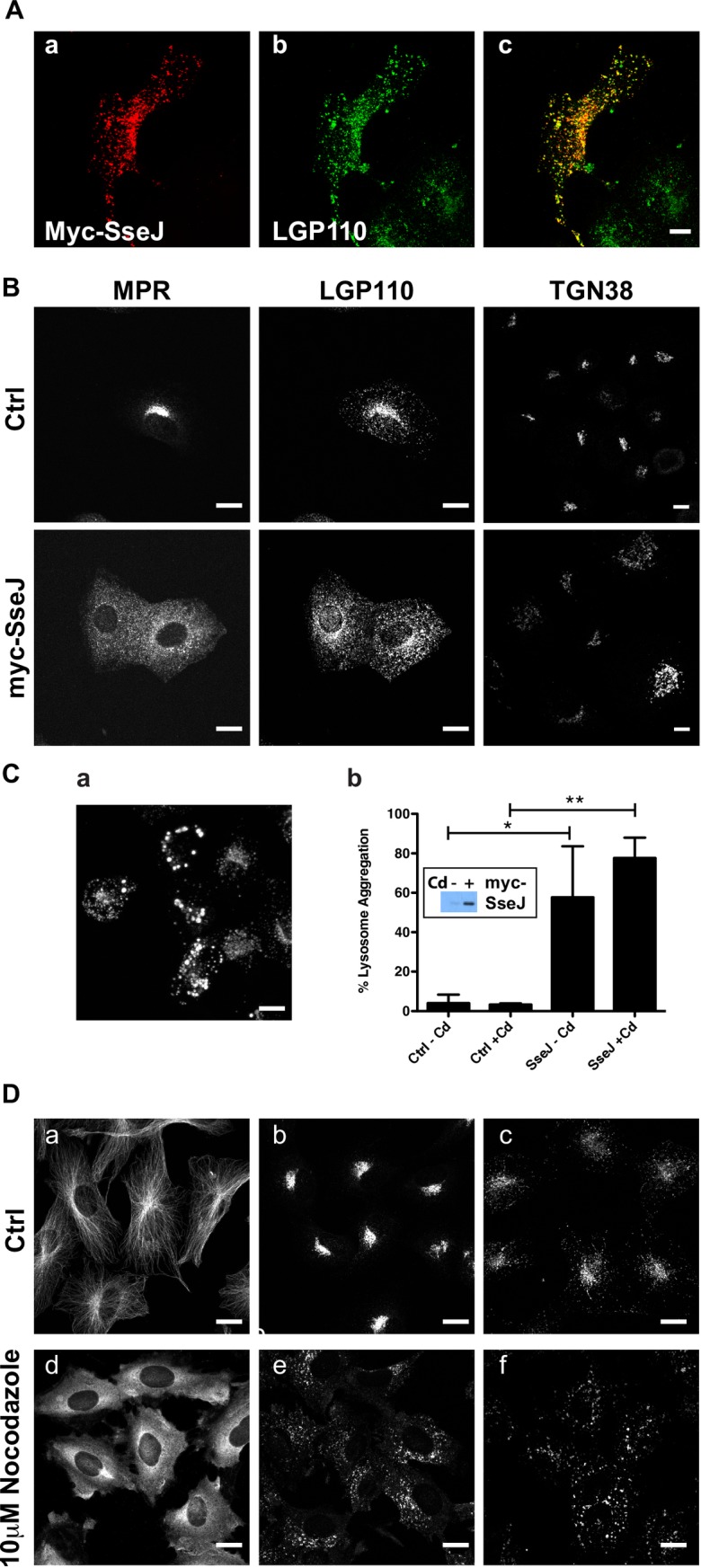
Re-distribution of late endocytic organelles in cells expressing SseJ. (A) NRK cells expressing myc-SseJ were double labelled with anti-myc (a) and anti-lysosome glycoprotein 110 (Lgp110; b) followed by fluorescently labelled secondary antibodies. Panel c is the merged image of panels a and b, co-localisation is shown by yellow. (B) Control (Ctrl) NRK cells or NRK cells expressing myc-SseJ (myc-SseJ) were immuno-labelled for the mannose 6-phosphate receptor (MPR), Lgp110 and *trans*-Golgi network 38 (TGN38) followed by fluorescently-labelled secondary antibodies to visualise the late endosomes, lysosomes and *trans*-Golgi network respectively. (C) Aggregation of Lgp110 in NRK cells expressing SseJ for 24h (a). Quantification of cells showing aggregated lysosomes after induction of SseJ production with cadmium (Cd) (b). Expression of myc-SseJ protein -/+ Cd is shown by the western blot insert (b). (D) NRK cells were immunolabelled for microtubules (-alpha-tubulin; a,d), lysosomes (lgp120;b,e) and late endosomes (cation-independent mannose 6-phosphate receptor; c,f) in control cells (ctrl) or after cells had been treated with 10μM nocodazole for 1h. Scale bars represent 10μm.

SseJ is one of several virulence proteins secreted by *Salmonella*’s T3SSs into the host’s cytoplasm directly from the bacteria [15]. *Salmonella* strains lacking SseJ are attenuated in replication [16–19] indicating that SseJ is crucial for bacterial intracellular replication. SseJ was then expressed in mammalian cells. In this case, we used Normal Rat Kidney (NRK) cells since they show good spatial resolution between endocytic vesicles and in particular between late endosomes and lysosomes. Late-endocytic organelles are poorly resolved by light microscopy in HeLa cells, which are often used for *Salmonella* infection studies. Constitutive protein production of SseJ was found to cause cell death so myc-tagged SseJ (myc-SseJ) was expressed under the control of a metallothionein promoter allowing for inducible *sseJ* expression upon the addition of cadmium. Immunofluorescence demonstrated that myc-SseJ localises to lysosomes (Fig 2A) as has previously been reported [19]). Moreover there was a dramatic re-distribution of late endocytic organelles with both late endosomes and lysosomes becoming less peri-nuclear and more peripherally distributed (Fig 2B). The *trans*-Golgi marker TGN38 was observed to occupy a larger area of the cell (Fig 2B), but in general cells were flatter with an increased surface area (on average the cell surface area went from 591μm^2^ to 2,057μm^2^ upon SseJ expression). Ectopic SseJ protein production can cause globular membranous compartments (GMCs) [19] and indeed when *sseJ* expression was induced for 24 h, lysosomes were seen to aggregate as observed by LGP120 (rat equivalent of LAMP1) staining (Fig 2C). The metallothionein promoter regulating *sseJ* expression is slightly leaky due to the presence of trace amounts of heavy metals in the tissue culture media, which explains why the lysosomes are partially aggregated in transfected cells before cadmium addition (Fig 2C panel b). The re-distribution of organelles is observed when the cytoskeleton is perturbed [20] and indeed when the microtubule polymerisation inhibitor nocodazole was added to cells, late endocytic organelles re-distributed in a manner similar to that observed with SseJ expression (Fig 2D).

### SseJ alters microtubule dynamics

**Fig 3:**
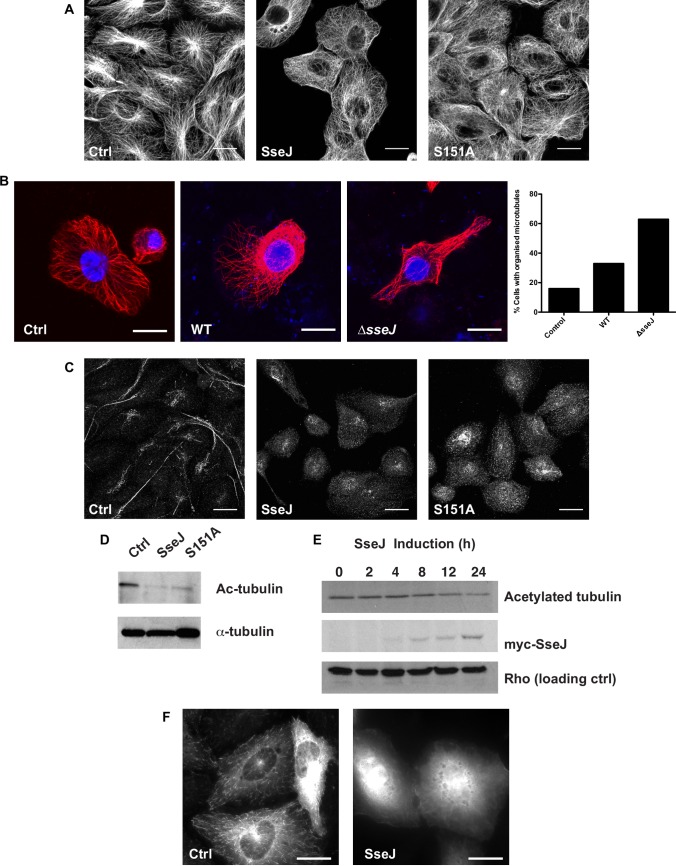
Microtubules are disrupted in cells expressing SseJ. (A) Control (Ctrl) NRK cells and cells expressing myc-SseJ (SseJ) or myc-SseJ-S151A (S151A) were fixed and the microtubules visualised using anti alpha-tubulin antibodies and fluorescently-labelled secondary antibodies. Bars = 10μm. (B) J774.2 mouse macrophages were either uninfected (Ctrl) or infected with WT or Δ*sseJ Salmonella* Typhimurium for 24h before fixing. The DNA (blue) was visualised using DAPI and the microtubules (red) were visualised as in A. Bars = 20μm. Quantification of the number of cells showing an organised microtubule network under each condition is shown (n = 1, scoring 100 cells per condition). (C) Cells as in A were fixed and de-tyrosinated alpha-tubulin (Glu-tubulin) visualised by immunolabelling using anti Glu-tubulin antibodies and fluorescently-labelled secondary antibodies. Bars = 10μm. (D) Cells as in A were lysed and lysates immunoblotted for acetylated-alpha-tubulin (Ac-tubulin) and alpha-tubulin. (E) myc-SseJ production was induced in NRK cells up to 24h. Lysates were generated and western blotted for myc-SseJ, acetylated-tubulin and Rho (pan specific). (F) NRK cells (Ctrl) and those expressing *sseJ* (SseJ) were transfected with a plasmid encoding EB3-tdTomato. EB3-tdTomato was visualised live, 24h later, on a spinning disc confocal microscope. Images represent a single time frame. Bars = 10μm.

To assess whether the re-distribution of organelles was related to changes to the cytoskeleton the microtubules were visualised in cells expressing SseJ or a mutant SseJ (SseJ S151A). SseJ has homology to the GDSL-like lipolytic enzyme family [21] and shows deacylase, phospholipase and glycerophospholipid-cholesterol acyltransferase (GCAT) activity [22–24]. Ser151 in SseJ is the middle serine in a GDSLS motif, which is present in GCAT enzymes and mutation of this residue reduces SseJ’s deacylase activity by 5 fold [22]. SseJ-S151A still localises to the *Salmonella* containing vacuole and *Salmonella* induced filaments (Sifs) [25] are still visible in a SseJ-S151A mutant strain but the bacteria show reduced virulence [22]. The microtubules, in both WT and mutant-SseJ expressing cells, became disorganised with no clear microtubule organising centre (MTOC; Fig 3A). In J774.2 macrophages the majority of cells don’t have clear microtubules emanating from a MTOC unless they have flattened out on the culture vessel surface (Fig 3B). Co-cultures of bacteria and J774.2 macrophages causes the macrophages to flatten out and under these conditions the microtubule network becomes more visible. However, a loss of organised microtubules, emanating from a clearly defined MTOC, was seen in mouse macrophages infected with WT *Salmonella* but not in cells infected with Δ*sseJ Salmonella* (Fig 3B). Typically, Δ*sseJ Salmonella* induced a four-fold increase in visible microtubules emanating from the MTOC compared to control cells, but WT *Salmonella* only induced a two-fold increase (Fig 3B). Unlike nocodazole that completely disrupts tubulin polymers (Fig 2D), cells expressing SseJ still show some tubulin polymers albeit in a dis-organised manner. Long-lived, stable microtubules are de-tyrosinated, resulting in the exposure of a glutamate residue (Glu-tubulin), and acetylated [26, 27]. Cells were then examined for the presence of Glu-tubulin (Fig 3C). In cells expressing both WT and mutant SseJ protein there was a reduction in Glu-tubulin immunolabelling compared to control cells. Furthermore, there was a reduction in acetylated-tubulin (Fig 3D) in WT and mutant *sseJ* expressing cells. The reduction in acetylated-tubulin corresponded to the time of induction of *sseJ* expression (Fig 3E). SseJ protein production was induced with 10μM cadmium and the metal can alter the cytoskeleton [28, 29] but we saw no effect of cadmium on the cytoskeleton in NRK cells without *ssej* expression (all control cells in Fig 3 are in the presence of 10μM cadmium chloride). Together these data suggested that long-lived microtubules had been de-stabilised in cells expressing SseJ, but some un-organised microtubules could still be observed. When cells were transfected with a plasmid encoding GFP-CLIP-170, a protein that binds to the growing ends of microtubules, and visualised by live cell microscopy, no CLIP-170 movement could be observed in cells expressing SseJ (S1 Movie) compared to control cells (S1 Movie). Similar data were obtained with EB3-tdTomato, another microtubule plus-end binding protein, and single images of EB3-tdTomato transfected cells show the EB3 on the end of microtubules in control cells but no visible incorporation of the EB3 onto microtubules in cells expressing SseJ (Fig 3F). So whilst there was a reduction in long-lived microtubules, as assessed by Glu-tubulin and acetylated-tubulin, there was no dynamism in the remaining microtubules.

### SseJ binds both RhoA and RhoC

**Fig 4:**
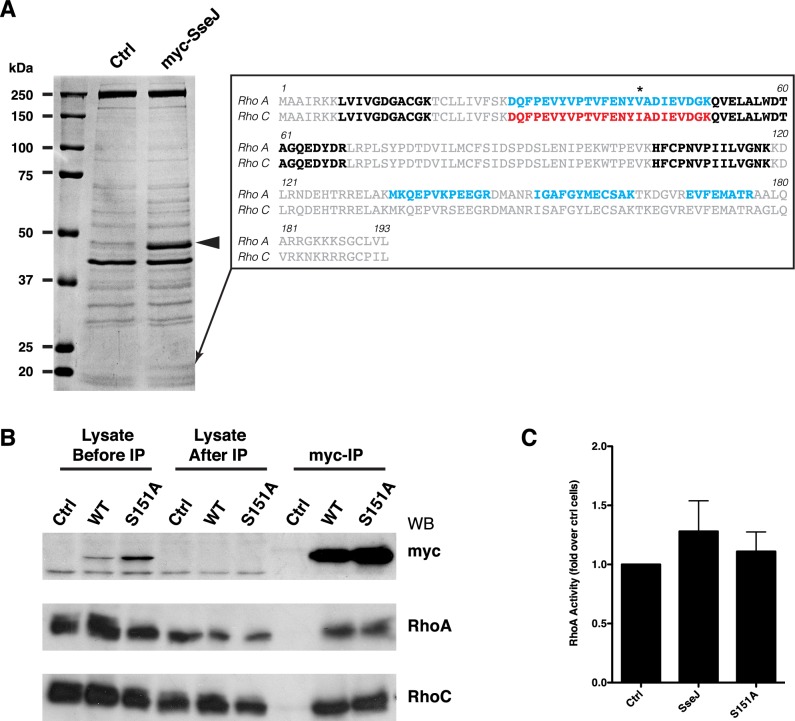
SseJ binds GTPases RhoA and RhoC. (A) Anti-myc antibody was covalently attached to sepharose and myc-SseJ was immunoprecipitated from control (Ctrl) NRK cells or NRK cells expressing myc-SseJ (myc-SseJ). Proteins bound to the beads were eluted and subjected to SDS-PAGE and the gel stained with coomassie (shown). SseJ is indicated by an arrowhead. A band at ≈21kDa specifically found in the SseJ immunoprecipitation was excised and sequenced by mass spectroscopy and identified both RhoA and RhoC. Peptides identified are shown by the insert with peptides common to both RhoA and RhoC shown in bold, peptides unique to RhoA shown in blue and peptides unique to RhoC shown by red. Only a single peptide was unique to RhoC (highlighted by an asterisk). (B) Experiments as shown in A, including cells expressing myc-SseJ(S151A), were repeated and western blotted for myc, RhoA and RhoC. Western blots show 1/10^th^ of the input before and after the immunoprecipitation and the total eluate from the immunoprecipitations. (C) The activity of RhoA was measured by ELISA, on extracts from control cells and cells expressing myc-SseJ or myc-SseJ (S151A) mutant. Data are means ± SD, n = 8.

Rho proteins are small GTPases that are primarily associated with modifying the actin cytoskeleton, but they can effect cell polarity and microtubules [30]. SseJ can interact with both RhoA or RhoC [31, 32], with GTP-bound RhoA activating SseJ’s lipase activity [32]. SseJ has only previously been shown to bind RhoA or RhoC separately. Large scale immunoprecipitations of SseJ from cells overexpressing SseJ identified both RhoA and RhoC having bound to SseJ under experimental conditions where the GTPases were in their GDP-bound form (Fig 4A), with WT and SseJ-S151A binding Rho proteins with equal ability (Fig 4B). These experiments indicate that SseJ can bind either RhoA or RhoC in the presence of each other when neither protein is in a limiting amount. Although we have no evidence, it is unlikely that SseJ is binding both RhoA and RhoC simultaneously. Using an ELISA we found, as has been reported [32], that SseJ did not increase the levels of activated (GTP-bound) RhoA (Fig 4C).

## Discussion

In this study, we aimed to understand how *Salmonella* can survive intracellularly by uncovering *Salmonella* effector molecules that can manipulate membrane trafficking events. Manipulation of membrane traffic may disrupt late-organelle biogenesis, including lysosomes, and therefore provide conditions that enable the bacteria to replicate. We hypothesised that a *Salmonella* T3SS effector molecule may manipulate membrane trafficking in yeast to the same extent as mammalian cells given that the delivery of molecules to the vacuole/lysosome are conserved. Using an unbiased screen we identified SseJ, which is a T3SS effector protein, that caused a membrane trafficking defect in yeast (Fig 1). This is the first demonstration that SseJ causes changes to membrane trafficking in eukaryotes. The powerful yeast screen led us to examine the distribution of organelles in mammalian cells, expecting them to be perturbed. Indeed, organelles no longer localised to the MTOC (Fig 2) and this observation could be related to changes to the microtubules (Fig 3). We further showed that SseJ can bind to both RhoA and RhoC and whilst others have shown that RhoA can regulate the GCAT activity of SseJ [32] this is the first report to prove the hypothesis that SseJ alters the cytoskeleton [33].

How might SseJ alter the cytoskeleton? Whilst Rho proteins are well known to alter the actin cytoskeleton they can also alter the stability of microtubules via Diaphanous-related formins (DRFs) [34]. RhoA-mDia1/2 can stimulate microtubule stabilisation with an increase in Glu-tubulin, precisely how this is achieved is unknown, and it is possible that if SseJ recruits active Rho proteins to the lysosome then the RhoA-mDia1/2 balance may be disrupted leading to changes in the microtubules. Whilst we did not observe an increase in Glu-tubulin we did see static microtubules. Although the binding of SseJ to RhoA or RhoC has been documented, our data show for the first time that SseJ can bind RhoA or RhoC when both proteins are present and neither are in limited amounts i.e. SseJ does not preferentially bind RhoA and then RhoC (Fig 4). This does raise the possibility that SseJ may have differential effects through both RhoA and RhoC, with differences between RhoA, RhoB and RhoC well documented [35]. So whilst RhoA-GTP can stimulate the GCAT activity of SseJ [32], the binding of RhoC to SseJ may affect the microtubules. RhoC is reported to have a higher affinity for the kinases Rho-associated coiled-coil containing kinases (ROCK) and Citron kinase compared to RhoA [35]. MAP2/Tau proteins stabilise microtubules and inhibit depolymerisation (reviewed by [36]), an effect seen in SseJ expressing cells, and MAP2/Tau proteins can be phosphorylated by numerous kinases including ROCK [35, 37]. The effects of MAP2/Tau phosphorylation are yet to be determined, but there is a precedence for microtubule regulation by Rho proteins via DRFs and kinases such as ROCK [38]. Expression of *sseJ* before *Salmonella* infection reduces Sif formation [19], which can be explained by the fact that a dynamic cytoskeleton is required for phagosome maturation [39]. Additionally, whereas SseJ-S151A has reduced GCAT activity [22] the effects on the microtubules are still seen in the S151A mutant suggesting that the GCAT activity is separate from the microtubule effect, though we can’t rule out that there is still enough residual GCAT activity in cells over-expressing *sseJ*.

SseJ has been shown to interact indirectly with another T3SS effector protein, SifA [31]. Δ*sifA* mutants escape the phagosomal vacuole but not if a double *sifA sseJ* mutant is made, implying that loss of the integrity of the phagosomal membrane is dependent on SseJ [19]. SifA and SseJ are sufficient to cause endosome tubulation [31] and certainly SifA is required for endosome tubulation [40, 41]. With SifA found to bind to RhoA, and SKIP, which is a kinesin binding protein, it was hypothesised that RhoA, SseJ, SifA and SKIP regulates endosome tubulation along microtubules [31]. However, studies have shown that Δ*sseJ Salmonella* show endosomal tubulation implying that SseJ is dispensable for endosome tubulation in a background where all the other secreted effector proteins are expressed [42, 43].

*Salmonella* induced endosomal tubules or *Salmonella* induced filaments (Sifs) are initially dynamic but become stabilised (>8h after cell infection; [42] and this stabilisation could correspond to the changes that we see in the dynamics of the microtubules, given that SseJ is secreted from *Salmonella* within 4 h [17]. It is has been known for a long time that lysosomes can form tubules [44, 45] and that microtubules regulate the distribution of lysosomes [46] and their tubular morphology [7]. Although SseJ is dispensable for the formation of Sifs in infected cells, SseJ may aid in stabilising the Sifs that do form. Why would this be advantageous to the *Salmonella*? Endosome fusion and delivery of endocytosed material to lysosomes can occur at the end of lysosome tubules [47] and the curvature of the membrane at the tip of a tubule is likely to be more fusogenic with endocytic vesicles compared to a larger, more-rounded phagosomal membrane [48]. By reducing microtubule de-polymerisation this allows *Salmonella* to promote tubular lysosomes (endosomal tubules), in conjunction with other proteins such as SifA, increasing fusion events with endosomal vesicles carrying in nutrients from the extracellular environment. Rho GTPases are a common target of bacterial pathogens [49, 50] and further work is required to determine whether SseJ’s effect on cellular microtubules is mediated through RhoA or RhoC.

## Materials and methods

### Reagents and antibodies

Chemical reagents were of laboratory grade. Anti-c-myc (9E10) antibodies were purified from 9E10 hybridoma tissue culture supernatants (Developmental Studies Hybridoma Bank). Anti rat LGP110 (580), anti-mouse cation-independent mannose 6-phosphate receptor (MPR; 1001) and anti-rat TGN38 (2F7.1) were kind gifts from J. P. Luzio (University of Cambridge, UK). Anti alpha-tubulin (T-9026) was from Sigma, anti-glu-tubulin was from Synaptic Systems, anti-acetylated tubulin (D20G3), rabbit monoclonal anti-RhoA (67B9) and anti-RhoC (D40E4) were from Cell Signalling.

### Yeast strains

BHY10 and BHY11 haploid yeast strains expressing CPY-Inv [51] and BHY10 ΔVPS10::TRP1 were a kind gift from Dr. M. Seaman (University of Cambridge). For the screen BHY10 and BHY11 were mated on YPD agar plates, diploid yeast (BHY12) picked from SC–Lys,-Ade plates and then maintained on YPD agar plates.

### *Salmonella* genomic library generation

Chromosomal DNA was isolated from stationary phase *Salmonella* Typhimurium strain 14028 [52]. DNA was partially digested with Sau3AI for 1h at 37°C. DNA was electrophoresed on a gel, and the region corresponding to ≈0.8–5 kb was excised and the DNA purified. pVT-100 U [53] a gift from Dr. K. Bowers (UCL, UK), was linearised with BamHI and then de-phosphorylated using calf intestinal phosphatase. DNA was ligated into linearised pVT-100U using T4 DNA ligase and transformed into NEB 10-beta competent *E*. *coli* (High Efficiency). Ampicillin-resistant colonies (≈0.5x10^6^) were scraped, and plasmid DNA prepared (Qiagen midiprep).

### Constructs

SseJ was cloned from *S*. Typhimurium DNA by PCR. Primers were used to append a myc-tag to the SseJ PCR product along with 15bp regions of homology to the destination vector to allow for homologous recombination using In-Fusion cloning (Invitrogen). The myc-tagged SseJ DNA was inserted into the HindIII restriction enzyme site of the ΔpMEP4 vector [54] by homologous recombination. The S151A mutant was made by QuikChange site-directed mutagenesis (Stratagene) of the myc-SseJ construct as per the manufacturer’s instructions.

### Invertase (Inv) assays

The *Salmonella* plasmid library was transformed into BHY12 yeast [55], and transformants were plated on synthetic complete medium without uracil (SC-Ura) plates with 2% (w/v) fructose. Carboxypeptidase-Y-invertase (CPY-Inv) assay, both quantitatively and qualitatively, is based on previous methodologies [10].

### FM4-64 staining

1ml of log-phase yeast were pelleted and then resuspended in 50μl YPD medium containing 40μM FM 4–64 (Molecular Probes). Yeast were incubated at 30°C for 15 min before the yeast were pelleted and washed with YPD media. Yeast in fresh YPD were then incubated for 30 min at 30°C. Yeast were pelleted and then resuspended in 1ml of H_2_0 and then aliquots directly visualised by confocal microscopy.

### Tissue culture and cell transfection

All cells were cultured in Dulbecco’s Modified Eagle’s Medium (DMEM), supplemented with 10% (v/v) FCS, 100U/L penicillin, 100mg/L streptomycin and 2mM L-glutamine, in a humidified atmosphere with 5% CO_2_. Cells were transfected with plasmid DNA using Fugene 6 (Roche Diagnostics) as per the manufacturer’s instructions. ΔpMEP4 transfected cells were selected with media containing 0.2 mg/ml hygromycin to generate a stable population of transfected cells and individual clones were selected and assessed for SseJ protein production. SseJ production was induced with 10 μM CdCl_2_ for 16-24h before analyses.

### J774.2 *Salmonella* infection

J774.2 cells were seeded onto glass coverslips and cultured for 48 h in antibiotic-free DMEM medium supplemented with 10% (v/v) FBS (heat inactivated to 56°C for 30 min) and 2 mM glutamine. *Salmonellae* (WT and ΔSseJ *Salmonella enterica* serovar *Typhimurium* strain 12023 were a kind gift from Prof. David Holden, Imperial College London). were cultured overnight in LB media with shaking at 30°C. An appropriate number of bacteria were taken to infect J774.2 cells at an MOI (multiplicity of infection) of 10 and resuspended in PBS. Bacteria were centrifuged onto cells at *80 x g* for 5 min and incubated for 1 h at 37°C to allow phagocytosis of bacteria. Monolayers were rinsed 3 times with DMEM to remove unbound bacteria, and the media replaced with DMEM containing 150 μg/ml gentamycin to kill extracellular bacteria. The cells were cultured for a further hour, and washed with PBS. The media was then replaced with DMEM containing 10 μg/ml gentamycin, and cells cultured for 24 h to allow intracellular bacteria to grow. Cells were fixed with 4% formaldehyde in PBS for 20 min at room temperature and then processed for immunofluorescence.

### Immunofluorescence

Cells were fixed with 4% (w/v) formaldehyde in PBS for 20 min at 20°C. Cells to be immunolabelled for microtubules were rinsed with microtubule stabilising buffer (MTSB; 80mM PIPES, pH 6.8, 1mM MgCl_2_, 4mM EGTA) then incubated in MTSB containing 0.05% (w/v) saponin (Sigma S-4521) for 1 min then fixed with 2% (w/v) formaldehyde, 0.05% (w/v) glutaldehyde in MTSB for 20 min. Cells immunolabelled for Glu-tubulin were fixed with -20°C MeOH for 5 min at -20°C. All fixed cells were incubated for 10 min in 50 mM NH_4_Cl in PBS followed by 10 min in 0.2% (w/v) BSA in PBS containing 0.5% (w/v) saponin (PBS-BS). Cells were immunolabelled with primary antibodies in PBS-BS for 1h at 20°C. Cells were rinsed 3 x 5 min with PBS-BS and then incubated with fluorescent secondary-antibodies in PBS-BS for 30 min at 20°C. Cells were rinsed 3 x 5 min with PBS-BS before being mounted in Mowiol. Fluorescence was imaged using a Zeiss LSM510 confocal microscope. All images are maximum intensity z-projections unless otherwise stated.

### Cell lysates

Lysates were generated by rinsing cells with PBS and then scraping cells into ice cold lysis buffer (150 mM NaCl, 20 mM Tris, pH 8.0, 2 mM EDTA, 0.5% (v/v) NP-40). Lysates were left on ice for 10 min before removal of detergent insoluble material by centrifugation (16,400 *g*, 10 min, 4°C).

### Immunoprecipitation

9E10 antibody was coupled to Amino Link Plus resin (Pierce) following the manufacturer’s instructions. Small scale immunoprecipitations used 20 μl of resin and 250μg of cell lysate and samples were incubated for 2 h at 4°C with rotation. Resins were washed 3 x with lysis buffer and immunoprecipitated proteins eluted using IgG gentle elution buffer (Pierce) and analysed by SDS-PAGE. Large scale immunoprecipitations used 12 x T75 flasks and 2ml of anti-myc resin.

### Mass spectroscopy

Proteins in gel bands were reduced with DTT and alkylated with iodoacetamide before digestion with modified porcine trypsin (Promega). Digests were dissolved in 4-hydroxy-alpha-cyano-cinnamic acid and analysed by positive-ion MALDI-MS/MS using a Bruker ultraflex III. Spectra were submitted to Mascot MS/MS ions search against the NCBI database.

### RhoA activity assays

Active RhoA in cell lysates was assessed by ELISA using a RhoA activity assay (RhoA G-LISA; Cytoskeleton, Inc) as per the manufacturer’s instructions.

## Supporting information

S1 Movie NRK cells (WT) or expressing SseJ were transfected with either GFP-CLIP170 (kind gift of Folma Buss, University of Cambridge) or EB3-tdTomato (a kind gift from Dr Anne Straube, University of Warwick) and 24 hours later imaged on an Andor Spinning Disc Confocal Microscope. Images were collected with 200ms exposures and a 800ms delay between exposures, giving 1 frame per second.
(MOV)
